# The impact of using cannabis during pregnancy on the infant and mother: An overview of systematic reviews, evidence map, targeted updates, and *de novo* synthesis

**DOI:** 10.1111/ajo.13916

**Published:** 2024-12-19

**Authors:** Zachary Munn, Danielle Pollock, Jennifer Stone, Sabira Hasanoff, Andrea Gordon, Carrie Price, Michael Stark, Timothy Hugh Barker

**Affiliations:** ^1^ Health Evidence Synthesis, Recommendations and Impact (HESRI), School of Public Health University of Adelaide Adelaide Australia; ^2^ JBI, School of Public Health University of Adelaide Adelaide Australia; ^3^ The Robinson Research Institute University of Adelaide Adelaide Australia; ^4^ Albert S. Cook Library Towson University Maryland USA

**Keywords:** female, fetal development, hallucinogens, humans, infant, newborn, systematic reviews as topic

## Abstract

**Background:**

Cannabis use during pregnancy is becoming more prevalent. While numerous studies have explored the relationship of cannabis use during pregnancy and outcomes for mothers and infants, uncertainty remains regarding the impact of cannabis use on pregnancy complications and later‐life outcomes for offspring.

**Aims:**

To produce a summary of the short and long‐term effects of prenatal cannabis exposure on fetal growth and development, neonatal conditions, later‐life, and maternal outcomes.

**Materials and Methods:**

An overview of systematic reviews, an evidence and gap map, targeted updates of previous reviews, and *de novo* evidence synthesis was conducted. The databases searched include PubMed (National Center for Biotechnology Information); MEDLINE (Ovid); Embase (Ovid) and CINAHL with Full Text (EBSCO). Assessment of risk of bias was conducted in duplicate for all studies. Relevant studies were coded and are presented as an evidence and gap map. Where possible, meta‐analyses were conducted with a narrative synthesis of the results. Primary studies and systematic reviews examining the relationship between cannabis consumption in pregnancy and the effect on fetal/child development, antenatal, and obstetric outcomes during pregnancy were eligible for inclusion.

**Results:**

There were 89 studies/reviews eligible for inclusion in this review. There was a potentially harmful impact of prenatal cannabis exposure on all fetal growth and development outcomes, some neonatal outcomes, some later‐life outcomes, and some maternal outcomes. The evidence regarding other neonatal conditions, later‐life, and maternal outcomes was mixed.

**Conclusions:**

The evidence suggests cannabis should be avoided during pregnancy.

## INTRODUCTION

The prevalence of cannabis (marijuana) use has become widespread among the general population for medical and recreational purposes, with an estimated prevalence of 2 to 5% in Australia, Canada and USA.^1^, ^2^, ^3^ Recent changes in legislation surrounding cannabis consumption has brought about significant shifts in usage trends and perceptions of associated risks,^1^, ^4^ leading to a decreased perception of cannabis as harmful and a simultaneous increase in its use.^5^ This evolving landscape introduces a challenge in understanding the risks and consequences of cannabis use, particularly among pregnant women, potentially influencing their decisions about using cannabis during pregnancy.^2^, ^5^, ^6^, ^7^, ^8^

It has been found that some pregnant women turn to cannabis^1^, ^9^ for relief from various conditions, including pre‐existing health concerns and challenges related to pregnancy. A notable motivation for this choice is the belief that cannabis can alleviate nausea during early pregnancy, as well as manage appetite and mood.^10^ This belief rests on the assumption that the associated risks for both the expectant mother and the developing fetus are minimal or negligible.^11^, ^12^, ^13^ However, the scientific community remains divided on this issue, with ongoing research needed to provide clearer guidance.

Numerous studies of varying quality have explored the relationship between cannabis use during pregnancy and its effects on both the mother and the infant. Tetrahydrocannabinol (THC), the psychoactive component of cannabis, is believed to be transferred from the mother to the fetus through the placenta during pregnancy.^8^, ^14^, ^15^, ^16^ This transfer raises concerns about potential developmental disruptions to the fetus as previous studies on humans and animals have found harmful effects.^3^ Furthermore, research has found harmful effects of cannabis on the person ingesting the drug.^17^ Despite these research efforts, uncertainty remains regarding the impact of maternal cannabis use on pregnancy complications and later‐life outcomes for offspring.^1^, ^7^, ^9^ Unlike the well‐established understanding of the effects of other substances like tobacco and alcohol, the effects of maternal and *in utero* cannabis use has not been appropriately collated and effectively communicated to policy makers, highlighting the need for concentrated efforts in this area.^1^

As such, clear and straightforward communication that accurately conveys the body of evidence related to the potential consequences of prenatal cannabis exposure on the mother, the fetus, and the child as they develop is crucial.^13^ Bridging this knowledge gap and ensuring effective communication to all relevant parties is essential for informed decision‐making by expectant mothers, healthcare providers, and policymakers. Our goal is to contribute to this effort by providing a comprehensive overview of the impact of cannabis use during pregnancy across a range of outcomes.

## REVIEW QUESTIONS

**Question 1:** For infants exposed prenatally to cannabis, what are the effects of cannabis on fetal development, neonatal withdrawal, birth outcomes, infant development, and child development (up to age 16 years)?

**Question 2:** For women who use cannabis in pregnancy, what are the effects of cannabis on antenatal/obstetric outcomes?

## MATERIALS AND METHODS

The protocol was registered with PROSPERO (CRD42023390292). An ethics statement is not applicable as this is a review of studies. To conduct this review in a rigorous and timely manner, an approach similar to ‘GRADE Adolopment’^18^ for adapting, adopting or developing new guidelines where none exist was applied. Initially, an overview of reviews approach was followed to identify the ‘best estimate of the effect’, which is defined as the association between maternal cannabis exposure and the prioritised outcomes of interest. This estimate was used for a particular outcome when a credible^19^ up‐to‐date systematic review (from 2018 onwards, in an attempt to include only recent evidence) reported the outcome. Certainty in these results was established using the Grading of Recommendations Assessment Development and Evaluation (GRADE) approach.^18^

Where multiple systematic reviews existed, the estimate that was deemed the most credible was used as the basis for a targeted update. Where multiple reviews existed assessing the same outcome and they were of similar credibility, the congruency of the results was assessed and, where needed, an updated or new meta‐analysis was developed.

Where new studies that reported an association between the exposure and the outcome of interest were identified, the best estimate from the systematic review was updated. Where studies report prioritised outcomes and no systematic reviews existed, we conducted our own synthesis of these results on studies post‐2018.

Furthermore, an evidence map of all studies from 2018 that met the inclusion criteria was developed. The following items were used to categorise the studies in EPPI‐Reviewer^20^ (Site Licence; Institute of Education, University of London): outcome domain, outcome measured, date of study, study location, study design.

### Inclusion criteria

#### Population and exposure

This review focuses on pregnant women and infants exposed to cannabis. It targeted studies clearly linking cannabis consumption (as the primary drug in cases of poly‐drug use) to fetal and child development. The scope included papers that reviewed poly‐drug consumption and concurrent tobacco smoking, in cases where the paper was able to comment on the quality of the evidence concerning cannabis despite concurrent use of other drugs or tobacco.

Studies exploring cannabis's effect on antenatal/obstetric outcomes during pregnancy were included. Cannabis may have been taken through any route of administration (eg smoked or ingested).

#### Comparator

No cannabis use during pregnancy.

#### Outcomes

Figure 3 lists all outcomes that contributed data organised into four outcome domains. A full list of prioritised outcomes is presented in an online repository (osf.io/6f8w3). These were informed by the National Academy of Sciences review into prenatal cannabis exposure^1^ and supplemented by the author team and other literature.

#### Types of studies

Peer‐reviewed studies from The Organisation for Economic Cooperation and Development (OECD) countries published in the English language that specified an effect of cannabis exposure and its impact on fetal or child development or maternal antenatal and obstetric outcomes were included. Credible^18^, ^21^ systematic reviews/meta‐analyses from 2018 were included. The evidence map included all relevant studies from 2018 and informed targeted updates or *de novo* synthesis.

The following criteria was used to determine credible reviews:

- published in the past five years (2018 onwards)
- included a comprehensive search strategy of two or more databases
- included formal critical appraisal/risk of bias assessment of included studies
- where multiple credible reviews existed, those that had performed a meta‐analysis and those that had applied GRADE^22^ were preferenced.

Only randomised controlled trials, cohort studies, and case–control studies were eligible for the evidence map and targeted updates. Cross‐sectional studies, case reports, and case series were excluded due to their likelihood of only providing very low certainty evidence. Despite considering randomised controlled trials, none were found due to the nature of the questions. Other study designs excluded were editorials studies by anonymous authors, conference abstracts, commentaries, animal studies, studies of prevalence and qualitative research. Studies where the population was too narrow and that were not likely to be applicable (ie cannabis exposure in opioid‐dependent women only) were excluded.

### Search strategy

The search strategy was developed with the input of a health librarian and peer‐reviewed according to the Peer Review of Electronic Search Strategies (PRESS) Statement^23^ by another information scientist. An initial limited search of PubMed to identify relevant articles on this topic was undertaken. The terminology contained in the titles and abstracts of relevant articles and the related subject headings and index terms used to describe the articles were used to develop a full search strategy for PubMed. The search strategy, including all identified keywords and index terms, was adapted for each included database and/or information source, using Polyglot^24^ and with the aid of a health librarian. The search was limited to publication dates from January 1, 2018 through the search date of January 4, 2023. The full search strategies for major databases are available in an online repository (osf.io/6f8w3).

The databases searched included PubMed (National Center for Biotechnology Information); MEDLINE (Ovid); Embase (Ovid); and CINAHL with Full Text (EBSCO). In the protocol for this review, it was originally planned to supplement the database search with Epistemonikos and Google Scholar specifically for systematic reviews using the key terms ‘marijuana or cannabis’ and ‘pregnancy’. However, due to time and resource constraints, this supplemental search was not conducted.

### Study screening and selection

All identified citations were collated and uploaded into EndNote™ and duplicates removed. Studies were imported into the Deduplicator^25^ tool for additional deduplication, and then imported into Covidence^26^ for screening. Two or more independent reviewers screened titles and abstracts against the inclusion criteria. Potentially relevant studies were retrieved in full and assessed against the inclusion criteria by two or more independent reviewers in EPPI‐Reviewer. Disagreements that arose at any stage of the selection process were resolved through discussion or with an additional author. The results of the search and the study inclusion process is reported in full and presented in a Preferred Reporting Items for Systematic Reviews and Meta‐analyses (PRISMA 2020) flow diagram^27^ for the overview of reviews, evidence map and targeted updates.

## MAPPING THE EVIDENCE

Studies included at the full‐text stage of screening in EPPI‐Reviewer were then subjected to mapping and categorisation. The following items were used to categorise the studies: outcome domain, outcome measured, date of study, study location, study design.

### Assessment of methodological quality/critical appraisal

Studies were assessed for risk of bias using either the JBI Cohort or JBI Case–control tools.^21^, ^28^ One review author assessed the risk of bias, and this was double‐checked by another member. Where a credible systematic review existed, the original risk of bias assessments of the individual studies were applied where possible. Any disagreements that arose between the reviewers were resolved through discussion, or with an additional reviewer/s. Risk of bias was undertaken at the study level and modified for GRADE risk of bias considerations at the outcome level if needed when a study reported multiple outcomes and this had an impact on critical appraisal judgements.

### Data extraction

Extraction forms were tailored by the research team for systematic reviews and primary studies (see online repository; osf.io/6f8w3). The data extracted included specific details about the participants, concept, context, study methods and key findings relevant to the question/s. Data extraction forms were piloted by all members of the research team. One reviewer extracted data from the included evidence sources which was double‐checked by another reviewer. Disagreements in extractions were resolved through discussion, or with an additional reviewer.

### Data synthesis and meta‐analysis

Meta‐analyses were sourced from credible reviews. Where updates or *de novo* synthesis was needed, studies were pooled, where possible, in a meta‐analysis where two or more studies reported results for the same outcome in a format conducive to meta‐analysis using Review Manager 5 (RevMan5, Cochrane Training, London, England). Where there was only one study contributing data to a particular outcome for a comparison, a forest plot is still presented for consistency purposes and to facilitate interpretation of the data.

For dichotomous data, effect sizes were calculated as odds ratios and presented with 95% confidence intervals (CIs). Incidence rate ratios were calculated where incidence rates were reported. Weighted (or standardised) mean differences and their 95% CIs were calculated for analysis for continuous outcomes or outcomes assessed using scales. Adjusted estimates were preferred but unadjusted estimates were used if no adjusted estimates were available. Effect sizes reported as correlation or regression coefficients are discussed narratively due to the difficulties and (often) inappropriateness of combining these effect estimates. The choice of model (random or fixed effects) and method for meta‐analysis were based on the guidance by Tufanaru et al.^29^ To facilitate meta‐analysis, interquartile ranges were converted to standard deviations where needed and possible.^30^

The credible systematic reviews underpinning a best estimate of an effect for an outcome may have included studies of lower evidence than cohort or case–control studies. These estimates were still used even if they used lower levels of evidence; however, they were only updated with cohort or case–control studies.

For assessment of heterogeneity and publication bias, the *I* ^2^ was interpreted according to the thresholds and guidance in the Cochrane Handbook for Systematic Reviews of Interventions.^31^ For meta‐analyses with more than ten studies, funnel plots were developed to investigate the possibility of publication bias. All detailed meta‐analyses and forest plots are presented in the online repository. Narrative summaries of the results are presented in this report.

Subgroup analyses were performed where relevant data existed and presented potentially important differences in the population or exposure (eg concomitant tobacco or opioid exposure).

### GRADE

The GRADE approach^32^ was followed for grading the certainty of evidence and GRADE evidence profiles were created using GRADEpro GDT for each comparison for prognostic factors.^33^ Evidence from observational studies begins with a ‘high certainty’ rating. In the GRADE approach for establishing certainty in the evidence relating to the effect of an intervention, evidence from non‐randomised studies begins as low certainty. However, as a prognostic factor was being assessed, this evidence begins as high in line with guidance from the GRADE Working Group.^33^ The evidence profile presents the following information where appropriate: absolute risks for the exposed and control, estimates of relative risk, and a rating of the certainty of the evidence based on the risk of bias, indirectness, heterogeneity, imprecision and risk of publication bias of the review results. Unless otherwise specified, baseline/comparative risks come from the control event rate or averages, or baseline characteristics of the sample. The outcomes reported in the evidence profiles have been prioritised by the funders of this review.

## RESULTS

A summary of results is presented in Figure 3, along with a GRADE Certainty of the Evidence rating for each included outcome.

### Study inclusion

**Figure 1:**
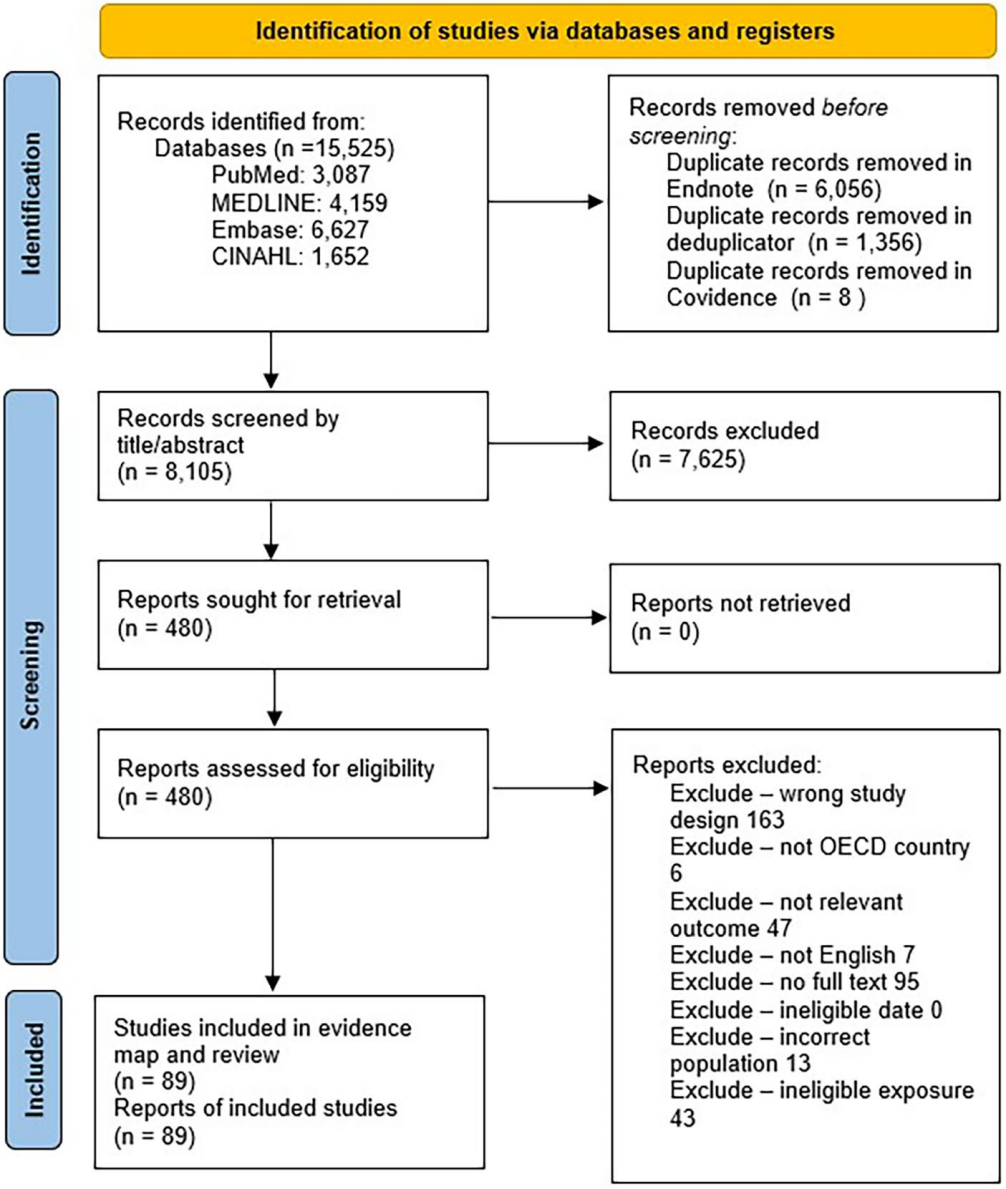
This PRISMA flow diagram depicts the number of documents identified through the search and screen process. There were 8105 citations identified for title and abstract screening in Covidence. There were 480 citations for full‐text review, with 391 studies excluded. A total of 89 reports were then included in this review.

Following the exclusion of duplicate citations in Endnote™ and then in the Deduplicator tool, 8105 citations were identified for title and abstract screening in Covidence. There were 480 citations for full‐text review, with 391 studies excluded. A total of 89 reports were then included in this review (Fig. 1).

### Methodological quality

The methodological quality of the studies substantially varied. In many studies, an issue identified was inadequate assessment of the exposure, as in many studies this was self‐reported as opposed to toxicology reports, urine tests or meconium testing. Another issue was not adequately addressing potential confounders in analyses. The full results of the study appraisals are available in the online repository.

### Characteristics of included studies

This review included 89 studies overall. Of these, 58 were cohort studies, nine were case–control studies and 22 were systematic reviews. Most of the primary studies were from the US (*n* = 50).

**Figure 2:**
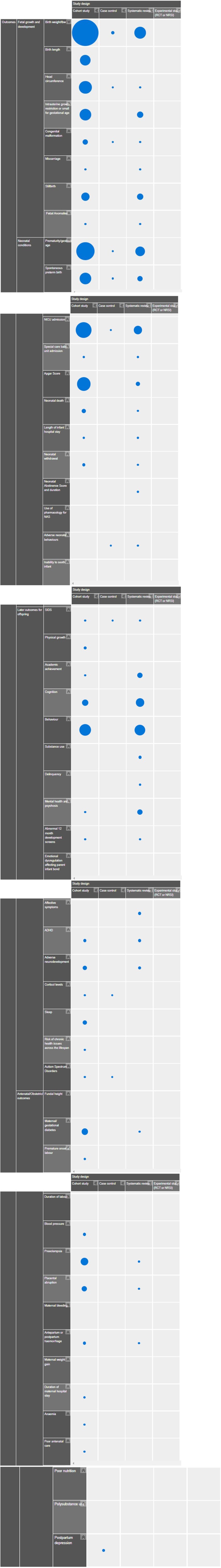
Static screenshot of the evidence gap map. Study counts: Birthweight/lbw **39;** Birth length **10;** Head circumference **15;** Intrauterine growth restriction or small for gestational age **17;** Congenital malformation **7;** Miscarriage **3;** Stillbirth **14;** Fetal anomalies **2**. Prematurity/gestational age **28;** Spontaneous preterm birth **17;** NICU admission **24;** Special care baby unit admission **2;** Apgar score **17;** Neonatal death **5;** Length of infant hospital stay **2;** Neonatal withdrawal **4;** Neonatal abstinence score and duration **1;** Adverse neonatal behaviours **2**. SIDS **3;** Physical growth **3;** Academic achievement **7;** Cognition **14;** Behaviour **21;** Substance use **3;** Delinquency **2;** Mental health and psychosis **7;** Abnormal 12‐month development screens **2;** Affective symptoms **3;** ADHD **6;** Adverse neurodevelopment **7;** Cortisol levels **3;** Sleep **4;** Risk of chronic health issues across the lifespan **2;** Autism spectrum disorders **2**. Maternal/ gestational diabetes **8;** Premature onset of labour **1;** Blood pressure **3;** Pre‐eclampsia **8;** Placental abruption **7;** Antepartum or postpartum haemorrhage **4;** Duration of maternal hospital stay **1;** Anaemia **1;** Poor antenatal care **1;** Postpartum depression **1**

The full details for the primary studies and credible reviews have been provided in the online repository. The full interactive evidence map is included in the online repository, with a static screenshot of the map provided in Figure 2.

### Findings of the review

**Figure 3:**
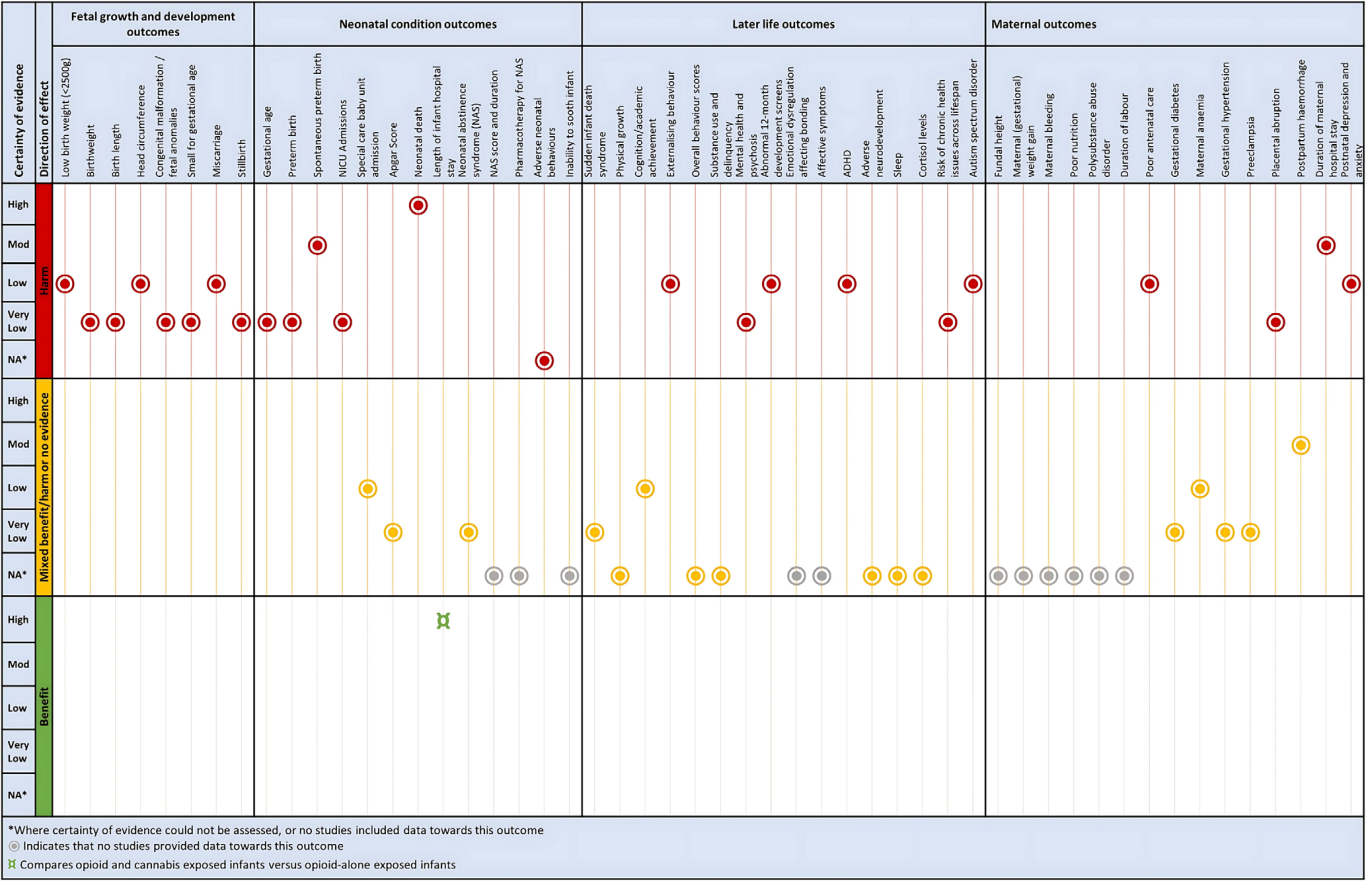
Overview of findings. Each outcome that contributes data is listed by the outcome domain in the top row. On the first column, the associated certainty of evidence rating is organised from highest level of certainty to lowest, or where certainty can't be assessed. It is further categorised into the direction of the effect, which can range from harm (red) to benefit (green).

An overview of the results for each outcome that contributed data is presented in Figure 3.

Full analyses (including meta‐analysis and GRADE evidence profiles) are available in an online repository (osf.io/6f8w3).

## FETAL GROWTH AND DEVELOPMENT OUTCOMES

For the domain of fetal growth and development, the evidence suggests a harmful impact on miscarriage, low birthweight, head circumference (low certainty), birthweight, birth length, congenital malformations, smallness for gestational age and stillbirth (very low certainty) (Table 1).

**Table 1:** Overview of findings for all outcomes

Outcome	No. of systematic reviews (credible reviews)	No. of primary studies	Result (exposed vs unexposed)
Fetal growth and development			
#Low birthweight (LBW)	9 (3 credible)	10 cohort studies	Significantly increased odds of LBW (odds ratio (OR) = 1.70, 95% CI: 1.44, 2.00)
#Birthweight (grams)	8 (3 credible)	13 cohort studies, and 2 case–control studies	Significantly decreased birthweight (mean deviation (MD) = −149.07 g, 95% CI: −197.19 g, −100.95 g)
#Birth length	None	10 cohort studies	Significant reduction in birth length (MD = −0.86, 95% CI: −1.30, −0.41)
#Head circumference	2 (1 credible)	11 cohort studies, and one case–control study	Significantly decreased head circumference (MD = −0.59, 95% CI: −0.65, −0.53)
#Congenital malformation / fetal anomalies	2 (1 credible umbrella review)	6 cohort studies, and 1 case–control study	Risk ratio (RR): significant increase in the risk of congenital malformation / fetal anomaly (RR = 1.27, 95% CI: 1.17, 1.38) OR: significant increase in the odds of congenital malformation / fetal anomaly (OR = 2.02, 95% CI: 1.91, 2.14)
#Small for gestational age (SGA)	5 (2 credible)	7 cohort studies	Significant increase in the odds of SGA (OR = 1.60, 95% CI: 1.50, 1.70)
#Miscarriage	None	1 cohort study	Significant increase in the risk of miscarriage/stillbirth (adjusted OR (aOR) = 12.1, 95% CI: 1.03, 141.8)
#Stillbirth	6 (2 credible)	7 cohort studies	RR: significant increase in the risk of stillbirth (RR = 1.63, 95% CI: 1.27, 2.09) OR: significant increase in the odds of stillbirth (OR = 1.29, 95% CI: 1.07, 1.56)
Neonatal conditions			
#Gestational age	6 (1 credible)	15 cohort studies and 1 case–control study	Significant decrease in gestational age (MD = −0.20 weeks; 95% CI: −0.35, −0.05)
#Preterm birth and spontaneous preterm birth	6 (4 credible)	8 cohort studies	OR preterm birth: significant increase in the odds of preterm birth (OR = 1.36, 95% CI: 1.29, 1.44) OR spontaneous preterm birth: significant increase in the odds of spontaneous preterm birth (OR = 1.80, 95% CI: 1.68, 1.93) RR preterm birth: significant increase in the risk of preterm birth (RR = 1.26, 95% CI: 1.12, 1.42) RR spontaneous preterm birth: no association between prenatal cannabis exposure and risk of spontaneous preterm birth (RR = 1.21, 95% CI: 0.76, 1.93)
#Neonatal intensive care unit (NICU) admissions	4 (4 credible)	14 cohort studies and 1 case–control study	Significant increase in the odds of infants being admitted to the NICU (OR = 1.55, 95% CI: 1.25, 1.91)
#Special care baby unit admission	1 (1 credible)	1 cohort study	No association (aOR = 1.7, 95% CI: 0.7, 4.0)
#Apgar score	3 (3 credible)	11 cohort studies	Apgar score at 1 min: no association (MD = −0.23, 95% CI: 0.70, 0.23) Apgar score at 5 min: no association (MD = −0.04, 95% CI: −0.14, 0.05) Apgar score <7 at 5 min (RR): no association (RR = 1.21, 95% CI: 0.81, 1.80) Apgar score <7 at 5 min (OR): significant increase in the odds (OR = 1.50, 95% CI: 1.10, 2.05) Apgar score <7 at 1 min: no association (OR = 1.0, 95% CI: 0.8, 1.25) Apgar score <4 at 5 min: significant increase in the odds (RR = 1.95, 95% CI: 1.64, 2.32)
#Neonatal death	1 (1 credible)	4 cohort studies	Significantly associated with neonatal death (OR = 1.79, 95% CI: 1.42, 2.27)
#Length of infant hospital stay	None	1 cohort study	The odds of a prolonged hospital stay were lower for prenatal cannabis exposure (OR = 0.68; 95% CI: 0.54, 0.86)
#Neonatal withdrawal / neonatal abstinence syndrome	1 (1 credible)	3 cohort studies	No significant association (OR = 0.83; 95% CI: 0.67, 1.02)
#Adverse neonatal behaviours	None	1 case–control study	Self‐regulation: B = −0.185 (standard error (SE): 0.091, *P* = 0.043) Handling: B = 0.112 (SE: 0.061, *P* = 0.066) Attention: B = −0.185 (SE: 0.207, *P* = 0.371) Lethargy: B = 0.058 (SE: 0.051, *P* = 0.256)
Later‐life outcomes			
#Sudden infant death syndrome (SIDS)	1 (not credible)	1 case‐cohort study	Full adjusted was an OR of 1.74 (95% CI: 0.29, 10.6; *P* > 0.500)
#Physical growth	None	4 cohort studies	Not statistically significant
#Cognition/ academic achievement	10 (1 credible)	10 cohort studies.	Mixed results
#Behaviour (overall and externalising)	9 (3 credible)	11 cohort studies	Mixed results
#Mental health and psychosis	5 (0 credible)	2 cohort studies	Marijuana use after knowledge of pregnancy was associated with increased offspring psychosis proneness when adjusting for covariates (beta coefficient 1.41, 95% CI: 0.34, 2.48; *P* = 0.010) Psychotic like experiences in children aged 10: not statistically significant (1.37, 95% CI: 0.9, 2.08)
#Abnormal 12‐month development screens	1 (0 credible)	1 cohort study	Adjusted relative risk of 1.90 (95% CI: 0.92, 3.91)
#Attention‐deficit/hyperactivity disorder (ADHD)	3 (0 credible)	3 cohort studies	Hazards ratio (HR) of 1.14 (95% CI: 1.02, 1.28), indicating an association with prenatal cannabis exposure and ADHD
#Adverse neurodevelopment	3 (0 credible)	3 cohort studies	Developmental or behavioural diagnosis: children with progressive myoclonic epilepsy (PME) did not differ from those without PME in terms of the likelihood of having a developmental or behavioural diagnosis from a health professional (aOR: 0.95, 95% CI: 0.05, 17.21) Cerebral palsy (CP): exposure to cannabis and other intrauterine drugs provided a similar risk to developing CP (OR: 1.86, 95% CI: 0.65, 5.28). Atypical neurological exam at 6–9 months: unadjusted OR of 1.69 (95% CI: 0.54, 5.26) Atypical neurological exam at 12 months: unadjusted OR of 3.09 (95% CI: 0.76, 12.53)
#Autism spectrum disorders (ASD)	None	2 cohort studies	Increased risk in the exposed group (HR 1.51, 95% CI: 1.17, 1.96)
Maternal outcomes			
#Poor antenatal care	None	1 cohort study	Pregnant people who consume cannabis are more likely to initiate care later (risk difference (RD) = 0.30, 95% CI: 1.15, 1.45)
			Cannabis‐exposed pregnant people were less likely to receive adequate prenatal care (aOR = 0.59, 95% CI: 0.48, 0.73)
			The cannabis‐exposed pregnant group had a lower likelihood of receiving a postpartum healthcare visit (OR = 0.65, 95% CI: 0.51, 0.82)
#Gestational diabetes	2 (0 credible)	6 cohort studies	No association between prenatal cannabis use and gestational diabetes (OR = 0.91, 95% CI: 0.66, 1.25)
#Maternal anaemia	None	1 cohort study	Non‐significant finding of prenatal cannabis use and maternal anaemia (adjusted relative risk = 1.15, 95% CI: 0.93, 1.40)
#Gestational hypertension	None	3 cohort studies	No significant association between prenatal cannabis use and an increased risk of gestational hypertension (OR = 1.06, 95% CI: 0.99, 1.14)
#Pre‐eclampsia	1 (0 credible)	7 cohort studies	No association between prenatal cannabis use and risk of pre‐eclampsia (OR = 0.93, 95% CI: 0.84, 1.04)
#Placental abruption	2 (0 credible)	5 cohort studies	A fixed effects meta‐analysis found an association between exposure to prenatal cannabis and increased risk of placental abruption (OR = 1.78, 95% CI: 1.62, 1.95)
#Postpartum haemorrhage	1 (0 credible)	2 cohort studies	No association between cannabis use within pregnancy and postpartum haemorrhage (OR = 1.04, 95% CI: 0.99, 1.09)
#Duration of hospital stay	None	1 cohort study	Postnatal stay 3–6 days: Those exposed to cannabis prenatally were more likely to be discharged from hospital after 3–6 days (aOR = 0.97 95% CI: 0.95, 0.98)
			Postnatal stay >7 days: Significant association
			Pregnant people exposed to cannabis were more likely to stay longer (aOR = 1.17, 95% CI: 1.11, 1.23)
#Postpartum depression and anxiety	None	1 cohort study	Anxiety: pregnant people who continued to use cannabis had a significantly higher risk of a higher generalised anxiety score than those who had never used cannabis (aOR = 2.55, 95% CI: 1.31, 4.96)
			Depression: those who continued cannabis were more likely to have higher Edinburgh Postnatal Depression Scale scores compared with those who had never used (aOR = 2.75, 95% CI: 1.43, 5.29)

## NEONATAL OUTCOMES

For neonatal outcomes, a harmful impact of cannabis consumption was found on the following outcomes: neonatal death (high certainty), spontaneous preterm birth (moderate certainty), gestational age and neonatal intensive care unit (NICU) admissions (very low certainty evidence). There was mixed, equivocal or no evidence for special baby care unit admission, neonatal abstinence syndrome (NAS), Apgar scores, use of pharmacotherapy for NAS, and adverse neonatal behaviours (Table 1).

## LATER‐LIFE OUTCOMES

For later‐life outcomes, the evidence suggests a harmful impact on externalising behaviour, abnormal 12‐month development screens, attention‐deficit/hyperactivity disorder and autism spectrum disorders (low certainty) and mental health and psychosis, and the risk of chronic health issues across the lifespan (very low certainty). There was mixed, equivocal or no evidence of sudden infant death syndrome, physical growth, cognitive/academic achievement, overall behaviour, substance use and delinquency, adverse neurodevelopment, sleep and cortisol levels (Table 1).

## MATERNAL OUTCOMES

For maternal outcomes, the evidence identified a harmful impact on the duration of maternal hospital stay (moderate certainty), poor antenatal care, postnatal depression and anxiety (low certainty) and placental abruption (very low certainty). There was mixed, equivocal or no evidence for fundal height, maternal weight gain, bleeding, nutrition, polysubstance abuse disorder, duration of labour, gestational diabetes, maternal anaemia, gestational hypertension, pre‐eclampsia, antepartum or postpartum haemorrhage (Table 1).

## DISCUSSION

This review included 89 studies that met the inclusion criteria across seven countries, investigating the short and long‐term effects of prenatal cannabis exposure on fetal growth and development, neonatal conditions, later‐life, and maternal outcomes. Summarising the main results of this review, the cumulative and overall weight of evidence indicates prenatal cannabis exposure has a harmful impact on most outcomes for both the mother and the offspring.

This review was able to address key questions regarding the impact of prenatal cannabis on a range of outcomes. Although studies to address most of the prioritised outcomes of interest were found, many outcomes were only informed from data from a small selection of these studies. Where statistically and clinically appropriate, results were combined across studies in a meta‐analysis, available in the online repository (osf.io/6f8w3). To enable this pooling, at times it was necessary to use data presented in the papers and convert these to a form suitable for pooling. Sometimes, the data were simply not accessible in a format that facilitated meta‐analysis. Where possible, the authors chose to combine studies in a meta‐analysis, despite the exposures and settings being somewhat different. Studies rarely distinguished different levels of cannabis intake and rarely discussed the timing of cannabis exposure. It was also difficult to determine (for some outcomes) the impact of co‐exposures such as tobacco use. Care must also be taken in the interpretation of the outcomes presented in this review, that report a significant effect, but have only been informed by the single study. The findings of this review have indicated that prenatal cannabis exposure is linked to increases in miscarriage, length of infant hospital stay, rates of poor antenatal care, and maternal anxiety and depression. However, these findings are only based on the results of a single cohort study, with no credible systematic reviews available. The impact of prenatal cannabis exposure on these outcomes requires further research effort, to better guide care and support.

The strengths of this review include its pragmatic approach and blend of relevant synthesis methods to ensure provision of the best available estimates of the impact of cannabis exposure. Modern evidence synthesis approaches were followed for establishing the certainty of the evidence by applying the GRADE approach for prognostic factors. Despite these strengths, this is a ‘restricted’ review^34^; therefore the authors made some design choices to expedite the review process. First, the review team did not search for unpublished studies, studies in languages other than English were excluded, and a search prior to 2018 was not undertaken. Where possible, the authors did assess for publication bias through visual inspection of funnel plots but this was only feasible for a small number of analyses given the lack of studies for the majority of outcomes. Where funnel plots were developed, publication bias was not detected. However, there remains the possibility that there are several studies potentially finding no association between cannabis and adverse outcomes that have not been published or were not identified during the search of this review.

In terms of the applicability of the results of this review, most studies were conducted in the US where there are potentially important differences in terms of legislation, cannabis use patterns and social demographics as compared to other countries. A further limitation of this review is that it does not include original studies not captured in credible reviews published earlier than 2018. However, this does suggest that the main conclusions are based on the most recent evidence and studies. This may increase the directness and applicability of this evidence given the changing patterns of cannabis consumption and the types and strength of cannabis available. Regardless of some of these applicability considerations, we believe there is a substantial body of evidence that warrants consideration, and these findings are applicable in many different contexts.

Mapping of the current state of the evidence helps to steer researchers in a productive direction and reduces redundant research efforts. The evidence and gap map conducted as part of this review identified many areas where there was mixed, equivocal or no evidence. Therefore, future research efforts in cannabis and pregnancy research could be focused on these areas identified in our review and discussed above, particularly maternal and later‐life outcomes.

## CONCLUSION

This review has identified a substantial body of evidence supporting the negative impact of prenatal cannabis exposure. Although the evidence is not of high certainty across all outcomes, overwhelmingly the authors of included studies advised against cannabis intake during pregnancy where possible. The findings of this review support that recommendation.

## FUNDING

This review is supported by a National Health and Medical Research Council (NHMRC) Ideas Grant (APP2011958).

This work is funded by the New South Wales (NSW) Ministry of Health Centre for Alcohol and Other Drugs and coordinated by the Sax Institute.

Zachary Munn is supported by a NHMRC Investigator Grant (1195676).

## DISCLAIMER

This review was an Evidence Check rapid review brokered by the Sax Institute for the NSW Ministry of Health.

This Evidence Check review was produced using the Evidence Check methodology in response to specific questions from the commissioning agency.

It is not necessarily a comprehensive review of all literature relating to the topic area. It was current at the time of production (but not necessarily at the time of publication). It is reproduced for general information and third parties rely upon it at their own risk.

## CONSTRAINTS ON PUBLISHING

None.

## AUTHOR CONTRIBUTIONS

Each author certifies that their contribution to this work meets the standards of the International Committee of Medical Journal Editors. ZM, DP, SH, TB, JS, THB: conceptualisation, data curation, formal analysis, investigation, methodology, project administration, resources, software, validation, visualisation, writing – original draft preparation, writing – review and editing. MS, CP, AG: conceptualisation, methodology, resources, supervision, validation, writing – review and editing.
